# Draft Genome Sequences of 19 Clinical Isolates of Candida auris from Hong Kong

**DOI:** 10.1128/MRA.00308-20

**Published:** 2021-01-07

**Authors:** Herman Tse, Alan K. L. Tsang, Yiu-Wai Chu, Dominic N. C. Tsang

**Affiliations:** a Microbiology Division, Public Health Laboratory Services Branch, Centre for Health Protection, Department of Health, Hong Kong SAR, China; Vanderbilt University

## Abstract

Candida auris is an emerging human pathogen associated with multidrug resistance and nosocomial outbreaks. We report the draft genome sequences of 19 C. auris isolates that were associated with a cluster of cases in a hospital in Hong Kong.

## ANNOUNCEMENT

Candida auris was first described in Japan in 2009 (1). Since then, C. auris infections and nosocomial outbreaks have been reported globally (2, 3). Of particular concern is the high rate of multidrug resistance, which has contributed to significant mortality among hospitalized patients suffering from invasive C. auris infections (4). In 2019, a cluster of C. auris colonizations occurred in a public hospital in Hong Kong and affected 15 patients.

Whole-genome sequencing for the isolates was performed for outbreak investigation. C. auris isolates were cultured from clinical specimens, including endotracheal aspirate, nasal swab, axilla swab, groin swab, and rectal swab specimens (Table 1). The isolates were grown overnight on blood agar at 37°C. Genomic DNA was extracted using the cetyltrimethylammonium bromide-based method (5), followed by library preparation using the Nextera XT kit (Illumina, CA) and sequencing on a MiSeq sequencer (Illumina). Paired-end reads were processed using Trimmomatic v0.38 (6) to remove low-quality bases and adapter sequences with the following parameters: ILLUMINACLIP, 1:30:10; LEADING, 10; TRAILING, 10; SLIDINGWINDOW, 4:15; and MINLEN, 30. Quality-trimmed reads were *de novo* assembled using SPAdes v3.13.1 with the parameters only-assembler, careful, and cov-cutoff auto (7). Ragout v2.1.1 was utilized for reference-assisted scaffolding against reference genomes of strains B11220 (GenBank accession no. GCA_003013715.2) and B11245 (GenBank accession no. GCA_008275145.1) (8). Contigs and scaffolds smaller than 500 bp were filtered and excluded from the draft assemblies.

**TABLE 1:** Characteristics and accession numbers of genomes of C. auris in the present study

Isolate	Patient	Isolation source	Genome size (Mb)	No. of reads	No. of contigs	No. of scaffolds	Scaffold *N*_50_ (bp)	Scaffold GC content (%)	Contig accession no.	Scaffold accession no.	Read accession no.
Cau1901	1	Endotracheal aspirate	13.05	3,292,066	1,986	362	243,019	45.06	CACTHW010000000	CACTHW030000000	ERR3503255
Cau1902	1	Pooled swab (nasal, axilla, and groin)	13.19	3,210,208	2,159	433	258,084	45.04	CACTGN010000000	CACTGN030000000	ERR3503256
Cau1903	1	Rectal swab	12.92	6,090,642	756	109	161,728	45.14	CACTGL010000000	CACTGL030000000	ERR3503257
Cau1904	2	Pooled swab (nasal, axilla, and groin)	12.85	5,160,246	1,581	238	233,558	45.06	CACTGM010000000	CACTGM030000000	ERR3503258
Cau1905	2	Pooled swab (axilla and groin)	12.95	3,797,138	1,685	278	241,804	45.05	CACTHC010000000	CACTHC030000000	ERR3503259
Cau1906	2	Nasal swab	12.88	5,245,386	1,424	348	235,153	45.05	CACTGS010000000	CACTGS030000000	ERR3503260
Cau1907	3	Skin swab (axilla and groin)	12.60	3,666,438	733	133	260,717	45.16	CACTHB010000000	CACTHB030000000	ERR3503261
Cau1908	4	Skin swab (axilla and groin)	13.61	1,979,612	2,376	707	308,895	45.07	CACTGV010000000	CACTGV030000000	ERR3503262
Cau1909	5	Skin swab (axilla and groin)	13.32	2,125,554	2,416	575	235,706	45.03	CACTHA010000000	CACTHA030000000	ERR3503263
Cau1910	6	Skin swab (axilla and groin)	13.05	5,992,296	1,842	330	204,007	45.06	CACTGU010000000	CACTGU030000000	ERR3503264
Cau1911	7	Skin swab (axilla and groin)	13.29	2,556,728	2,447	527	263,577	45.01	CACTGZ010000000	CACTGZ030000000	ERR3503265
Cau1912	8	Pooled swab (axilla and groin)	13.46	4,233,504	837	154	167,847	45.18	CACTGW010000000	CACTGW030000000	ERR3503266
Cau1913	9	Nasal swab	12.71	5,291,608	749	101	232,540	45.15	CACTGY010000000	CACTGY030000000	ERR3503267
Cau1914	10	Nasal swab	12.83	3,683,864	813	126	172,921	45.18	CACTGX010000000	CACTGX030000000	ERR3503268
Cau1915	11	Nasal swab	13.08	3,481,116	784	112	194,566	45.13	CACTGQ010000000	CACTGQ030000000	ERR3503269
Cau1916	12	Pooled swab (nasal, axilla, and groin)	13.19	5,254,784	909	126	199,353	45.17	CACTGR010000000	CACTGR030000000	ERR3503270
Cau1917	13	Pooled swab (nasal, axilla, and groin)	14.25	4,755,080	996	213	113,160	45.18	CACTGT010000000	CACTGT030000000	ERR3503271
Cau1918	14	Pooled swab (nasal, axilla, and groin)	13.28	5,222,686	758	100	192,279	45.17	CACTGP010000000	CACTGP030000000	ERR3503272
Cau1919	15	Pooled swab (nasal, axilla, and groin)	12.58	4,471,008	656	69	208,443	45.13	CACTGO010000000	CACTGO030000000	ERR3503273

The draft assemblies of these 19 isolates varied in length from 12.6 to 14.2 Mb, with a mean ± standard deviation scaffold count of 265 ± 180, GC content of 45.1% ± 0.06%, scaffold *N*_50_ value of 217 ± 44 kb, and coverage depth of 86-fold ± 26-fold for quality-trimmed reads. Summary statistics for individual assemblies are presented in Table 1.

**FIG 1:**
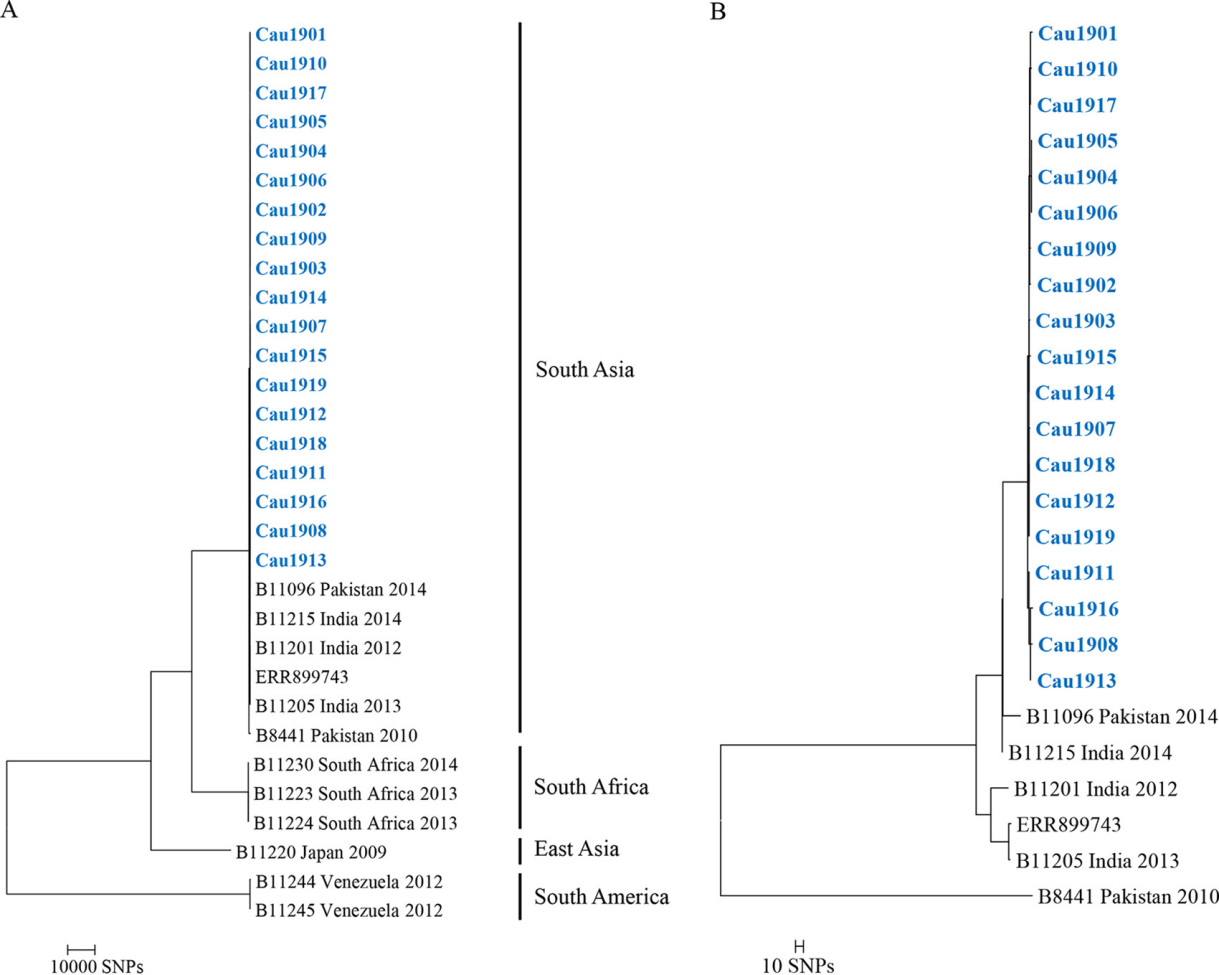
(A) Phylogenetic tree showing the genetic relationships among isolates representing four distinct clades. The isolates from the 15 patients are highlighted in blue. The scale bar indicates the number of SNP differences. (B) Phylogenetic tree showing the genetic relationships among isolates within the South Asia clade. The isolates from the 15 patients are highlighted in blue. The scale bar indicates the number of SNP differences.

Single nucleotide polymorphism (SNP) analysis was performed using Snippy v4.41 (https://github.com/tseemann/snippy). C. auris strain B8411 (GenBank accession no. GCA_002759435.2) was selected as the reference genome, and the raw reads of sequenced C. auris isolates from each of the four established clades, including strains B11215 (GenBank accession no. SRR3883446 [clade I/South Asia]), B11220 (GenBank accession no. SRR3883452 [clade II/East Asia]), B11223 (GenBank accession no. SRR3883455 [clade III/South Africa]), and B11244 (GenBank accession no. SRR3883465 [clade IV/South America]), were added to the analysis (9). A maximum likelihood phylogeny was constructed from the SNP data using IQ-TREE v1.6.9 with a GTR+gamma model and the fast option. All isolates are closely related to B11215, with 40 to 45 SNPs, which identifies them as strains of clade I/South Asia (Fig. 1A) (2). The maximum number of pairwise SNP differences between isolates is 13, suggesting a high degree of genetic relatedness (Fig. 1B). Our results are comparable to those of a previous study, which suggested a genetic distance of ≤12 SNPs between patients as being indicative of recent transmission (10).

Gene mutations associated with antifungal resistance, including a reported *ERG11* mutation, were observed (9, 11). The present work adds to the growing body of knowledge on this increasingly important human pathogen.

### Data availability.

The whole-genome sequencing project has been deposited in DDBJ/ENA/GenBank under the accession no. PRJEB34199. The accession numbers for the assembly and raw reads for individual isolates are provided in Table 1.
